# Fluorescence activated cell sorting followed by small RNA sequencing reveals stable microRNA expression during cell cycle progression

**DOI:** 10.1186/s12864-016-2747-6

**Published:** 2016-05-27

**Authors:** Vince Kornél Grolmusz, Eszter Angéla Tóth, Kornélia Baghy, István Likó, Ottó Darvasi, Ilona Kovalszky, János Matkó, Károly Rácz, Attila Patócs

**Affiliations:** 2nd Department of Medicine, Semmelweis University, Szentkirályi utca 46, 1088 Budapest, Hungary; “Lendület” Hereditary Endocrine Tumours Research Group, Hungarian Academy of Sciences, Semmelweis University, Szentkirályi utca 46, 1088 Budapest, Hungary; Department of Immunology, Eötvös Loránd University, Pázmány Péter sétány 1/C, 1117 Budapest, Hungary; 1st Department of Pathology and Experimental Cancer Research, Semmelweis University, Üllői út 26, 1085 Budapest, Hungary; Molecular Medicine Research Group, Hungarian Academy of Sciences – Semmelweis University, Szentkirályi utca 46, 1088 Budapest, Hungary; Department of Laboratory Medicine, Semmelweis University, Nagyvárad tér 4, 1089 Budapest, Hungary

**Keywords:** Fluorescence-activated cell sorting (FACS), Cell cycle, Dynamic expression, DNA staining, miRNA

## Abstract

**Background:**

Previously, drug-based synchronization procedures were used for characterizing the cell cycle dependent transcriptional program. However, these synchronization methods result in growth imbalance and alteration of the cell cycle machinery. DNA content-based fluorescence activated cell sorting (FACS) is able to sort the different cell cycle phases without perturbing the cell cycle. MiRNAs are key transcriptional regulators of the cell cycle, however, their expression dynamics during cell cycle has not been explored.

**Methods:**

Following an optimized FACS, a complex initiative of high throughput platforms (microarray, Taqman Low Density Array, small RNA sequencing) were performed to study gene and miRNA expression profiles of cell cycle sorted human cells originating from different tissues. Validation of high throughput data was performed using quantitative real time PCR. Protein expression was detected by Western blot. Complex statistics and pathway analysis were also applied.

**Results:**

Beyond confirming the previously described cell cycle transcriptional program, cell cycle dependently expressed genes showed a higher expression independently from the cell cycle phase and a lower amplitude of dynamic changes in cancer cells as compared to untransformed fibroblasts. Contrary to mRNA changes, miRNA expression was stable throughout the cell cycle.

**Conclusions:**

Cell cycle sorting is a synchronization-free method for the proper analysis of cell cycle dynamics. Altered dynamic expression of universal cell cycle genes in cancer cells reflects the transformed cell cycle machinery. Stable miRNA expression during cell cycle progression may suggest that dynamical miRNA-dependent regulation may be of less importance in short term regulations during the cell cycle.

**Electronic supplementary material:**

The online version of this article (doi:10.1186/s12864-016-2747-6) contains supplementary material, which is available to authorized users.

## Background

The fine-tuned mechanisms of cell cycle have always been in the focus of cancer research resulting in a better understanding and optimization of the action of several chemotherapeutic agents [1–3]. Both posttranslational modifications of proteins (e.g. protein-protein interactions, phosphorylations) and altered transcriptional activity of specific genes contribute to the tightly controlled regulation of the cell cycle [3]. Analysis of mRNA transcripts expressed in a cell cycle dependent manner using high-throughput screening methods have identified numerous genes used for differentiating malignant tumors from benign lesions [4–6]. These correlations were presumably due to the possibility that cell cycle dynamics accelerate in malignant tumors, resulting in a larger proportion of cells residing in S and G2 phases [4].

Former approaches for detecting transcripts expressed in a cell cycle dependent manner used several synchronization techniques for arresting the cell cycle at a certain point. Among others, serum starvation, double thymidine block, and thymidine-nocodazole block halted the cell cycle in cultured cells at G0, early S and M phases, respectively [4–6]. After removal of the synchronizing agent, time-course gene expression data followed by adequate bioinformatics analysis were used for identifying the cell cycle regulated transcripts [4–6]. However, several conflicting arguments have been raised concerning the usage of these synchronization procedures [7]. Statistical re-examination of a former study [6] surprisingly revealed that randomization of time-course gene expression data showed the same strong periodicity in the expression patterns as those obtained from the original synchonization experiment. Moreover, synchronization procedures in general [8], and DNA replication inhibitors such as thymidine in particular, result in the perturbance of cell cycle machinery, producing growth imbalance and unscheduled expression of cyclins [8, 9]. Additionally, cells may lose their synchronization relatively soon after the release from the synchronizing agent [5, 10] and only a subset of cells reenter the cell cycle after arrest [5, 11]. Therefore, a set of criteria has been introduced for the analysis of cell cycle dependent transcripts [7]. Accordingly, the expression pattern of a certain gene can be introduced as cell cycle dependent if (i) no inhibition or starvation method was used for synchronization, (ii) results are reproducible over several experiments (iii) results of additional methods other than microarrays (e.g. Northern blot, quantitative real-time polymerase chain reaction – qRT-PCR) support the findings (iv) expression patterns are confirmed in non-synchronized experiments (e.g. cells separated by size or DNA content) and (v) statistically robust analysis supports the results [7].

The regulation of the cell cycle in general and the cell cycle dependent transcriptional program in particular is the consequence of the precise interactions between cyclin-cyclin dependent kinase complexes and an oscillating network of transcription factors [12, 13]. Additionally, epigenetic mechanisms as microRNA-mediated regulations contribute to proper cell cycle regulation. MicroRNAs (miRNAs) are short, ~22 nt long noncoding RNA molecules regulating gene expression on the post-transcriptional level targeting the 3’ untranslated regions of mRNAs [14]. Extensive complementarity results in mRNA degradation, whereas in the case of short complementarity, transcriptional silencing is achieved by transcriptional repression [15]. Among other physiological functions, the importance of miRNA-dependent gene regulation has been confirmed in several key members of the cell cycle machinery [16–18], contributing to miRNA-dependent cell cycle changes [17, 19]. Altered expression of cell cycle-controlling miRNAs has been reported in neoplasms of various tissues [18, 20, 21]. Additionally, dynamic miRNA expression changes have been observed during exit from quiescent state due to serum reintegration into the culture medium of serum starved cells [22, 23]. In particular, elevated expression of E2F1 and E2F3 in response to mitogenic stimuli have been shown to enhance the expression of its transcriptional targets: hsa-let-7 and hsa-miR-16 family members [22]. Moreover, E2F1 has been shown to enhance hsa-miR-15 expression, which inhibits cyclin E, one of the key transcriptional targets of E2F1 [17]. Accordingly, it has been proposed that such feed-forward loops encompassing the E2F transcription factors, miRNAs and cyclins contribute to the fine-tuning of cell cycle regulation [17]. However, the potential dynamic miRNA expression changes between the cell cycle phases of actively cycling cells without any synchronization or serum shock procedures have not been thoroughly investigated.

Here, for the first time, we show that gene expression signature obtained from unperturbed cells sorted by fluorescence activated cell sorting (FACS) based on their DNA content at different phases of the cell cycle correlate well with former gene expression studies using synchronization methods. In addition to lower expression of cell cycle genes in different cell cycle phases, dynamic mRNA expression changes were found to be of greater amplitude in primary, untransformed fibroblasts as compared to those detected in cancer cell lines, reflecting the more precise cell cycle regulation in untransformed cells with lower proliferation characteristics. Using numerous high-throughput miRNA-screening methods, miRNA expression, unlike mRNA expression, was found to be quite stable throughout the cell cycle progression in various human cells.

## Results

### An optimized cell cycle sorting method successfully differentiates cell cycle phases in various cells

**Fig. 1:**
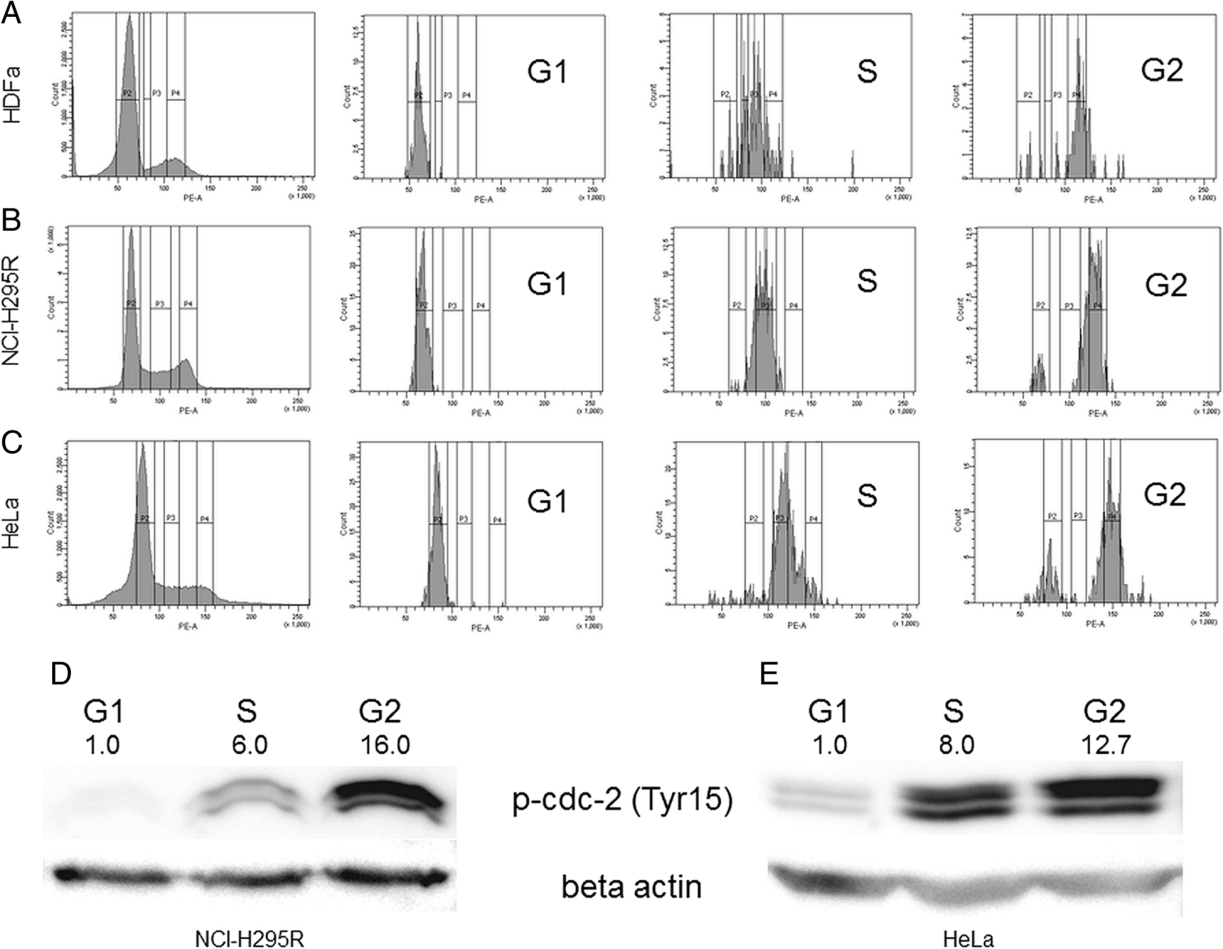
Fluorescence activated cell sorting (FACS) analysis of stained cells and validation of cell cycle sorted populations in HDFa, NCI-H295R and HeLa cells. Panels **a**-**c**: FACS analysis shows cell cycle distribution of stained cells before (first image) and after sorting to G1, S and G2 phases (second, third and fourth images, respectively; Panel **a**: HDFa, Panel **b**: NCI-H295R, Panel **c**: HeLa. Intervals of fluorescence intensity (shown as vertical bands) were defined to gate G1, S and G2 phases, respectively. Panels **d-e**: Western blot analysis confirms the high efficacy of cell cycle sort (Panel **d**: NCI-H295R, Panel **e**: HeLa). Density values of phospho(Tyr15)-CDC-2 were first normalized to β-actin loading control and were further normalized to G1 phase (displayed above each band)

Our optimal cell cycle sorting was able to differentiate cells residing in various cell cycle phases in all of the three cell types used (HDFa, NCI-H295R and HeLa cells) (Fig. 1, panel a-c). The purity of cell cycle sorted populations varied between cell types and cell cycle phases (Additional file 1: Table S1), but based on FACS reanalysis, these sorted cell populations were still more homogenous than cells obtained after synchronization procedures [5]. G1 phase was sorted most efficiently in all cell types with more than 95 % of purity in all cells. In NCI-H295R and HeLa cells, S phase cells showed more homogenous population as compared to cells in G2 phase.

Optimization of the cell cycle sort was needed for the sake of achiveving high purity in sorting without damaging or perturbing physiological cell functions. In particular, the determination of upper limit of sorting time, the use of a specialized sort medium and the immediate re-analysis of sorted cells contributed to our results.

### Protein expression change confirms the successful sorting

Tyr15 phosphorylation of CDC-2 protein is a tightly controlled event in cell cycle progression [24], thus the respective amounts of phospho (Tyr15)-CDC-2 provide a general hallmark for each phase. Western blot analysis performed on protein extracts of sorted NCI-H295R and HeLa cells showed the well known phosphorylation patterns of CDC-2 (Fig. 1, panel d-e), confirming the purity of cell cycle sorting on protein level as well.

### Gene expression profiling detects cell cycle regulated transcripts in FACS-sorted cells

**Fig. 2:**
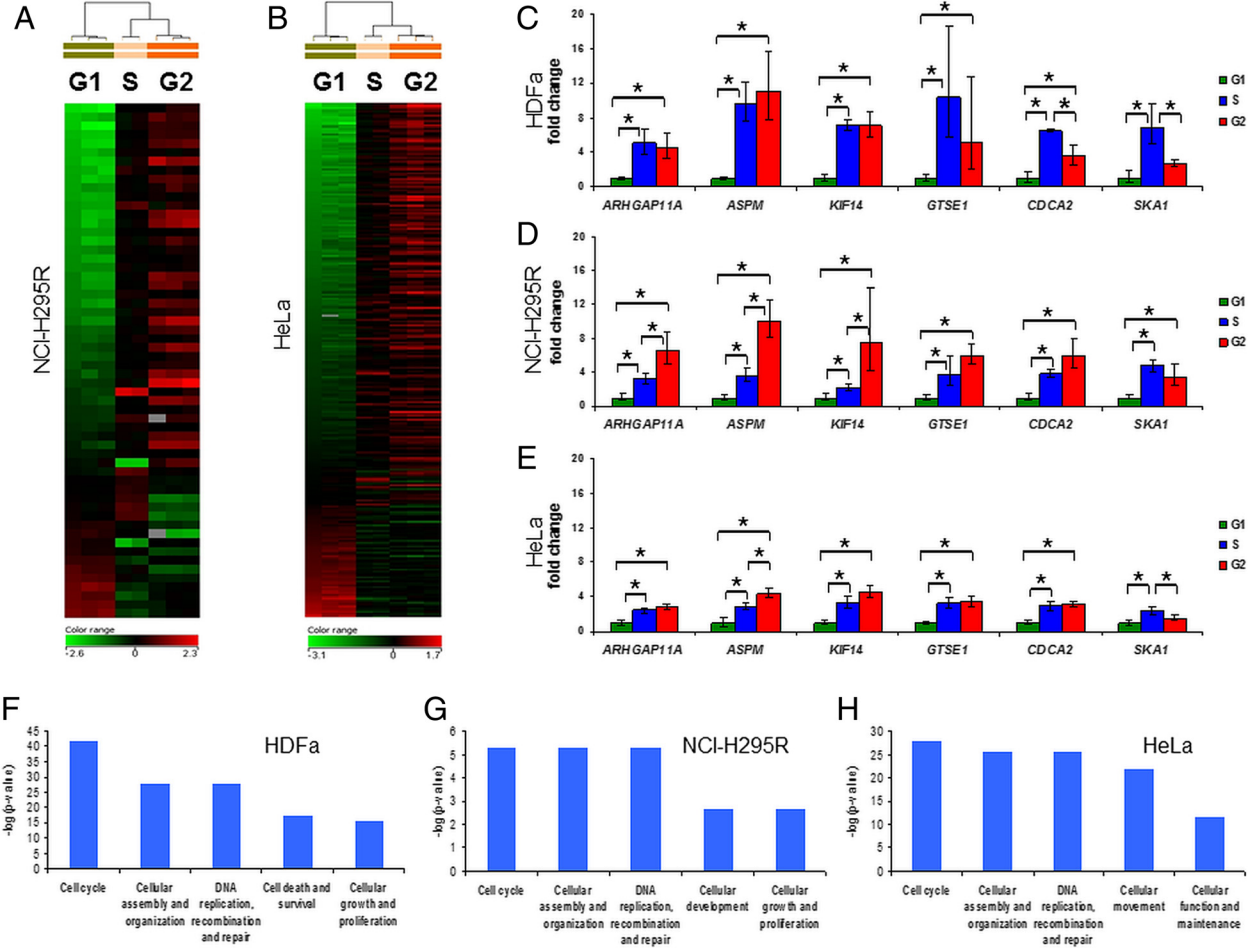
High throughput gene expression profiling, qRT-PCR validation and functional bioinformatics analysis of cell cycle dependent transcription in HDFa, NCI-H295R and HeLa cells. Panels **a-b**: Heat map of mRNA transcripts with significantly different expression between cell cycle phases (Panel **a**: NCI-H295R, Panel **b**: HeLa). Above the heat maps, hierarchical clustering upon differently expressed genes are also shown (additional information, including the list of gene symbols and descriptions can be found in Additional file 1: Table S3, panels B and C). Panels **c-e**: qRT-PCR validation of six genes chosen upon microarray analysis (Panel **c**: HDFa, Panel **d**: NCI-H295R, Panel **e**: HeLa). In each case ΔCt value was normalized to G1 phase (ΔΔCt) and was subjected to FC = 2^-ΔΔCt^ transformation. Error bars show standard deviation. Asterisks mark statistical significance (*p* < 0.05). Panels **f-h**: Molecular and cellular functions concerned by gene expression alterations in cell cycle phases. Δ(G2-G1) gene expression changes of significantly differently expressed genes (Panel **g**: NCI-H295R, Panel **h**: HeLa) or genes with fold change > 2 expression (Panel **f**: HDFa) were subjected to IPA core analysis. In each cell, the five most significantly concerned networks are shown. Significance threshold (*p* = 0.05) corresponds to –log(*p*-value) = 1.301. For additional molecular and cellular functions see Additional file 2: Figure S2

**Fig. 3:**
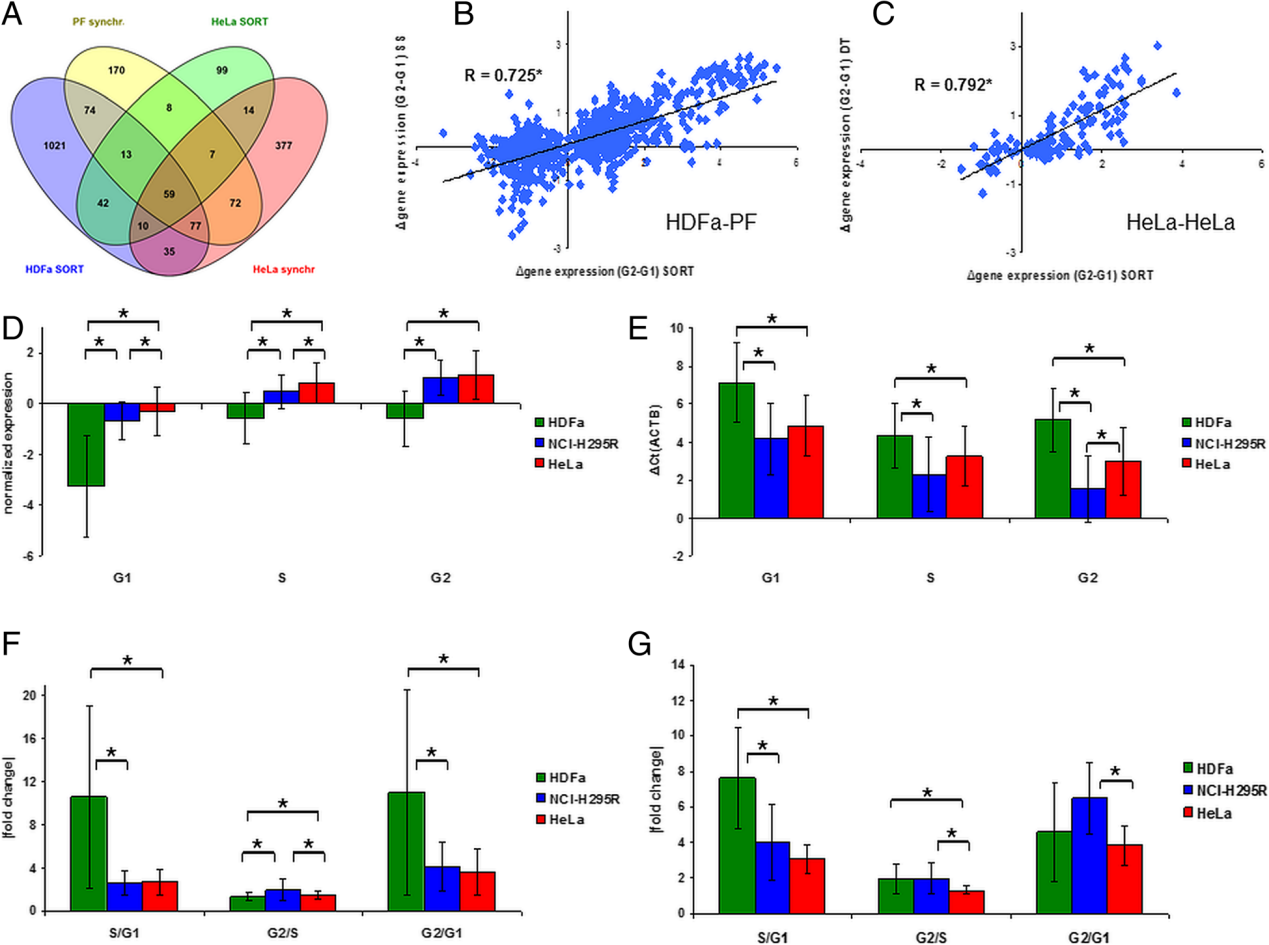
Comparison of cell cycle dependent gene expression observed by cell cycle sort and synchronization experiments and analysis of gene expression dynamics during the cell cycle in various cell types. Panel **a**: Venn diagram of cell cycle dependent genes detected in cell cycle sorted primary fibroblasts (HDFa SORT), in synchronized primary fibroblasts (PF synchr – data from [5]), in cell cycle sorted HeLa cells (HeLa SORT) and in synchronized HeLa cells (HeLa synchr – data from [4]). Intersections present the number of commonly found genes. For gene lists see Additional 1: Table S4. Panels **b-c**: Correlation analysis of gene expression differences using normalized expression values obtained from microarray experiments. Pearson’s correlation coefficient was calculated from Δ(G2-G1) expression changes detected by cell cycle sort and former synchronization experiments in primary fibroblasts (synchronization method: serum starvation – SS, Panel **b**) and HeLa (synchronization method: double thymidine block – DT, Panel **c**) cells. Correlation coefficients are displayed. Asterisks mark statistical significance (*p* < 0.05). For additional correlation calculations see Additional 2: Figures S3 and S4. Panels **d-e**: Analysis of normalized gene expression values of different cell types. Normalized gene expression of 127 cycling genes exported from microarray data (Panel **d**) and 10 cycling genes exported from qRT-PCR data (Panel **e**) were analyzed. Note the lower ΔCt(ACTB) values mean higher expression values. Error bars show standard deviation. Asterisks mark statistical significance (*p* < 0.05). Panels **f-g**: Analysis of mean gene expression fold change between cell cycle phases of different cell types. Absolute values of fold change of 127 cycling genes exported from microarray data (Panel **f**) and 10 cycling genes exported from qRT-PCR data (Panel **g**) of cell cycle sort experiments were analyzed. Error bars show standard deviation. Asterisks mark statistical significance (*p* < 0.05)

The quantity and quality of isolated RNA from sorted cells were sufficient to perform high throughput gene expression screening (Additional file 2: Figure S1, Additional file 1: Table S1). Gene expression profiling, followed by rigorous statistical analysis detected 55 mRNA transcripts in NCI-H295R cells (Fig. 2, Panel a, Additional file 1: Table S3, panel B) and 252 mRNA transcripts in HeLa cells (Fig. 2, Panel b, Additional file 1: Table S3, panel C) to be expressed in a cell cycle dependent manner. Note that the majority of detected gene expression changes share a common manner: expression rises as cell cycle proceeds. Additionally, clustering showed that S and G2 phases’ expressional patterns are closer to each other than to G1 phase. Statistical analysis of HDFa microarray data failed to detect genes with significantly altered expression, however, the functional bioinformatics analysis of the gene expression changes of greater than a twofold change (FC > 2, Fig. 2, Panel f, Additional file 1: Table S3, panel A) supports the concept that these changes strongly influence cell cycle progression. Moreover, the successful qRT-PCR validation of the chosen FC > 2 genes (Fig. 2, Panel c) and the significant correlation of gene expression changes of cell cycle sorted with former synchronization-based experiments in primary fibroblasts (Fig. 3, Panel b) further confirm the relevance of our approach.

Gene expression changes observed by qRT-PCR experiments of six genes chosen upon microarray analysis confirmed the microarray results in all of the three cells (Fig. 2, Panel c-e). Specifically, all the six genes chosen for the qPCR validation were present in the significant (NCI-H295R, HeLa) or FC > 2 (HDFa) lists. Moreover, *ARHGAP11A*, *KIF14* and *GTSE1* were previously found to be expressed in a cell cycle dependent fashion in primary fibroblasts [5] and HeLa cells [4], while *ASPM* and *SKA1* genes were found to be cell cycle regulated in primary fibroblasts [5]. The successful validation of these well-known cell cycle genes in all three cell types analyzed here further confirms our cell cycle sorting method.

Functional bioinformatics analysis was used to detect altered pathways based on our microarray results. As a further confirmation of our method, ”Cell cycle” ”Cellular assembly and organization” and ”DNA replication, recombination and repair” were the molecular and cellular functions most concerned by gene expression changes in all three cells (Fig. 2, Panel f-h).

### Comparison of cell cycle dependent expression between cell cycle sort and former synchronization based data

Several conflicting arguments arose on the applicability of synchronization procedures to define transcripts with cycling expression in unperturbed cells [7]. Therefore we aimed to compare expression changes between cell cycle phases detected by gene expression profiling in synchronization and cell cycle sort based experiments. Because synchronization based time course gene expression data in adrenocortical cell line have not been previously published, comparisons were made with primary fibroblasts and HeLa cells. Pearson’s method showed significant correlation between gene expression changes observed in synchronization based and cell cycle sort based experiments, confirming previous synchronization experiments by a synchronization-free method in unperturbed cells (Fig. 3, Panel a-c, Additional file 2: Figures S3 and S4, Additional file 1: Table S4).

Additionally, Gene Ontology (GO) Term analysis was performed on the HeLa cell cycle dependent transcriptional program to analyze the possible difference in biological processes affected by cell cycle sort and synchronization procedures. As both of cell cycle sort-based and synchronization-based results are only applicable in HeLa cells, we performed the analysis on three gene lists: genes unique to the HeLa cell cycle sort experiment (unique HeLa SORT), genes unique to the HeLa synchronization experiment (unique HeLa synchr) and the overlap between these two lists. All three lists were enriched with cell cycle-related processes; however, the overlap between the two experiments presented the most significant enrichment of cell cycle-associated biological processes, cross-validating important cell cycle genes detected by both the synchronization-based and cell cycle sort-based procedures. All the GO terms detected in the unique HeLa SORT list were detected in the overlap list, however, interestingly, five out of eight GO terms detected in the unique HeLa synchr list were unique to this list of genes, not being present in the analysis of the unique HeLa SORT or overlap gene lists (Table 1 and Additional file 1: Table S5).

**Table 1:** GO term analysis of the cell cycle dependent transcriptional program of HeLa cells

*GO Term*		*Gene count*	*p-value*	*Bonferroni-corrected p-value*
Panel A – genes unique to HeLa SORT experiment				
GO:0007049	cell cycle	19	4.62 × 10^−6^	3.32 × 10^−3^
GO:0000280	nuclear division	10	1.99 × 10^−5^	1.42 × 10^−2^
GO:0007067	mitosis	10	1.99 × 10^−5^	1.42 × 10^−2^
GO:0000087	M phase of mitotic cell cycle	10	2.29 × 10^−5^	1.64 × 10^−2^
GO:0048285	organelle fission	10	2.73 × 10^−5^	1.95 × 10^−2^
GO:0051301	cell division	11	3.41 × 10^−5^	2.43 × 10^−2^
GO:0007017	microtubule-based process	10	5.94 × 10^−5^	4.19 × 10^−2^
Panel B – genes unique to HeLa synchronization experiment				
GO:0006259	**DNA metabolic process**	15	1.20 × 10^−8^	7.35 × 10^−6^
GO:0007049	cell cycle	17	5.65 × 10^−8^	3.45 × 10^−5^
GO:0006281	**DNA repair**	10	2.23 × 10^−6^	1.36 × 10^−3^
GO:0006974	**response to DNA damage stimulus**	11	2.59 × 10^−6^	1.58 × 10^−3^
GO:0022403	cell cycle phase	11	6.53 × 10^−6^	3.99 × 10^−3^
GO:0006260	**DNA replication**	8	1.25 × 10^−5^	7.63 × 10^−3^
GO:0033554	**cellular response to stress**	12	1.67 × 10^−5^	1.02 × 10^−2^
GO:0000278	mitotic cell cycle	10	1.89 × 10^−5^	1.15 × 10^−2^
Panel C – overlap of genes of HeLa SORT and synchronization experiments				
GO:0022403	cell cycle phase	46	2.21 × 10^−49^	1.40 × 10^−46^
GO:0000279	M phase	43	1.12 × 10^−48^	7.09 × 10^−46^
GO:0000278	mitotic cell cycle	44	3.69 × 10^−48^	2.33 × 10^−45^
GO:0022402	cell cycle process	48	2.39 × 10^−46^	1.51 × 10^−43^
GO:0007049	cell cycle	52	6.85 × 10^−46^	4.33 × 10^−43^
GO:0007067	mitosis	37	3.87 × 10^−45^	2.45 × 10^−42^
GO:0000280	nuclear division	37	3.87 × 10^−45^	2.45 × 10^−42^
GO:0000087	M phase of mitotic cell cycle	37	7.76 × 10^−45^	4.90 × 10^−42^
GO:0048285	organelle fission	37	1.81 × 10^−44^	1.15 × 10^−41^
GO:0051301	cell division	32	3.01 × 10^−32^	1.90 × 10^−29^
GO:0007017	microtubule-based process	23	7.87 × 10^−21^	4.97 × 10^−18^
GO:0000226	microtubule cytoskeleton organization	17	7.86 × 10^−17^	7.02 × 10^−14^
GO:0007346	regulation of mitotic cell cycle	15	8.68 × 10^−14^	5.48 × 10^−11^
GO:0051726	regulation of cell cycle	19	1.68 × 10^−13^	1.06 × 10^−10^
GO:0007051	spindle organization	10	2.28 × 10^−12^	1.44 × 10^-.9^
GO:0007059	chromosome segregation	11	2.02 × 10^−11^	1.28 × 10^-.8^
GO:0010564	regulation of cell cycle process	12	2.90 × 10^−11^	1.84 × 10^-.8^
GO:0007010	cytoskeleton organization	18	1.70 × 10^−10^	1.08 × 10^-.7^
GO:0051783	regulation of nuclear division	9	6.98 × 10^−10^	4.41 × 10^-.7^
GO:0007088	regulation of mitosis	9	6.98 × 10^−10^	4.41 × 10^-.7^
GO:0000070	mitotic sister chromatid segregation	8	8.88 × 10^−10^	5.61 × 10^-.7^
GO:0000819	sister chromatid segregation	8	1.09 × 10^-.9^	6.89 × 10^-.7^
GO:0051276	chromosome organization	17	7.07 × 10^-.9^	4.47 × 10^-.6^
GO:0000075	cell cycle checkpoint	9	3.58 × 10^-.8^	2.26 × 10^-.5^
GO:0040001	establishment of mitotic spindle localization	5	6.00 × 10^-.8^	3.79 × 10^-.5^
GO:0051656	establishment of organelle localization	8	9.92 × 10^-.8^	6.27 × 10^-.5^
GO:0030071	regulation of mitotic metaphase/anaphase transition	6	1.12 × 10^-.7^	7.07 × 10^-.5^
GO:0007093	mitotic cell cycle checkpoint	7	1.23 × 10^-.7^	7.76 × 10^-.5^
GO:0051653	spindle localization	5	1.78 × 10^-.7^	1.13 × 10^-.4^
GO:0051293	establishment of spindle localization	5	1.78 × 10^-.7^	1.13 × 10^-.4^
GO:0048015	phosphoinositide-mediated signaling	8	5.37 × 10^-.7^	3.39 × 10^-.4^
GO:0008283	cell proliferation	14	6.95 × 10^-.7^	4.39 × 10^-.4^
GO:0051640	organelle localization	8	7.28 × 10^-.7^	4.60 × 10^-.4^
GO:0007052	mitotic spindle organization	5	1.14 × 10^-.6^	7.17 × 10^-.4^
GO:0051329	interphase of mitotic cell cycle	8	1.57 × 10^-.6^	9.90 × 10^-.4^
GO:0051325	interphase	8	1.90 × 10^-.6^	1.20 × 10^-.3^
GO:0007018	microtubule-based movement	8	2.92 × 10^-.6^	1.85 × 10^-.3^
GO:0000910	cytokinesis	6	2.93 × 10^-.6^	1.85 × 10^-.3^
GO:0033043	regulation of organelle organization	10	2.99 × 10^-.6^	1.89 × 10^-.3^
GO:0010948	negative regulation of cell cycle process	5	1.01 × 10^-.5^	6.36 × 10^-.3^
GO:0007094	mitotic cell cycle spindle assembly checkpoint	4	2.62 × 10^-.5^	1.64 × 10^-.2^
GO:0045841	negative regulation of mitotic metaphase/anaphase transition	4	2.62 × 10^-.5^	1.64 × 10^-.2^
GO:0031577	spindle checkpoint	4	3.47 × 10^-.5^	2.17 × 10^-.2^
GO:0045839	negative regulation of mitosis	4	3.47 × 10^-.5^	2.17 × 10^-.2^
GO:0051784	negative regulation of nuclear division	4	3.47 × 10^-.5^	2.17 × 10^-.2^
GO:0051439	regulation of ubiquitin-protein ligase activity during mitotic cell cycle	6	4.48 × 10^-.5^	2.79 × 10^-.2^
GO:0051438	regulation of ubiquitin-protein ligase activity	6	7.05 × 10^-.5^	4.36 × 10^-.2^
GO:0051303	establishment of chromosome localization	4	7.10 × 10^-.5^	4.39 × 10^-.2^
GO:0050000	chromosome localization	4	7.10 × 10^-.5^	4.39 × 10^-.2^

Gene lists from the Venn diagram (Fig. 3, Panel a) and Additional file 1: Table S4 were subjected to Gene Ontology term analysis. Genes unique to HeLa SORT experiment (HeLa SORT \ HeLa synchr - Panel A), unique to HeLa synchr experiment (HeLa synchr \ HeLa SORT – Panel B) and the overlap between these two lists (HeLa SORT ∩ HeLa synchr – Panel C) were the input gene lists, respectively

Gene count represents the number of genes from the input list being present in the corresponding GO term. Gene symbols are shown in Additional file 1: Table S5. Only statistical significant (Bonferroni-corrected *p*-value < 0.05) GO terms are presented. Bold lettered GO terms correspond to unique GO terms found only upon the analysis of either unique HeLa SORT or unique HeLa synchr gene lists

### Magnitude of gene expression alterations during cell cycle progression in untransformed and cancer cells

QRT-PCR validation of microarray experiments (Fig. 2, Panel c-e) indicated that gene expression changes might be characterized by different amplitudes in primary vs. cancer cells. Therefore, we analyzed the expression profiles and cell cycle dynamics of genes displaying altered expression between cell cycle phases in both primary untransformed (HDFa) and transformed cancer (HeLa) cells (127 genes present in both HDFa SORT and HeLa SORT gene lists, Fig. 3, Panel a). Significantly lower expression values were found in primary untransformed compared to cancer cells in G1, S and G2 phases as well (Fig. 3, Panel d-e). For the analysis of mRNA dynamics during cell cycle in untransformed and cancer cells, differences in mean fold changes of expression of genes commonly altered in HDFa and HeLa cell cycle sort experiments were calculated and evaluated (Fig. 3, Panel f-g). Among several significant alterations, a robust difference in mean fold change of gene expression was observed in G1/S transition between primary fibroblasts and cancer (NCI-H295R and HeLa) cells based on both microarray and qRT-PCR results. During the cell cycle, cycling genes had lower basal expression, but they demonstrated expression changes of significantly greater amplitude in primary non-transformed fibroblasts, than in transformed cancer (NCI-H295R and HeLa) cells.

### High-throughput screening of miRNAs differently expressed during the human cell cycle

**Fig. 4:**
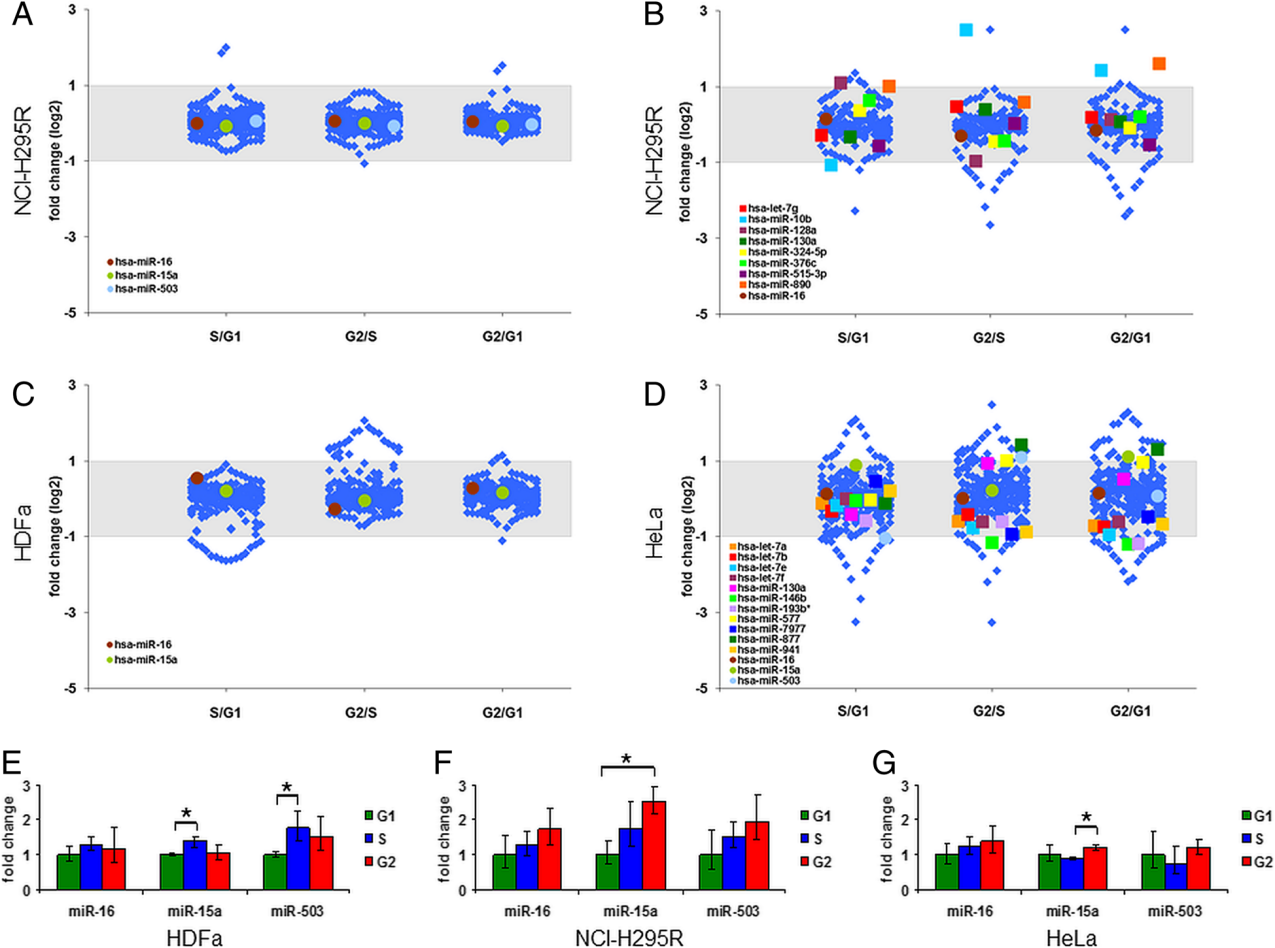
Analysis of cell cycle dependent miRNA expression by high-throughput screenings methods and individual qRT-PCR in HDFa, NCI-H295R and HeLa cells. Panels **a-d**: Fold change (log2) of miRNA expression in S/G1, G2/S and G2/G1 phases observed by microarray (Panel **a**: NCI-H295R cells, Panel **c**: HDFa cells), TaqMan Low Density Array (Panel **b**: NCI-H295R cells) and Illumina Small RNA Sequencing (Panel **d**: HeLa cells). Grey background corresponds to fold change ≤ 2 between cell cycle phases. Colored squares represent miRNAs with significantly different expression in cell cycle phases obtained by statistical analysis of high-throughput data. Colored circles represent some members of the hsa-miR-16 family, if available. For further high-throughput data of NCI-H295R cells (Illumina Small RNA Sequencing) see Additional 2: Figure S5. For qRT-PCR measurement data see Additional 2: Figure S6. Panels **e-g**: QRT-PCR measurements of hsa-miR-16 family members hsa-miR-16, hsa-miR-15a and hsa-miR-503 in HDFa (Panel **e**), NCI-H295R (Panel **f**) and HeLa (Panel **g**) cells. In each case ΔCt values were normalized to G1 phase (ΔΔCt) and were subjected to FC = 2^-ΔΔCt^ transformation. Error bars show standard deviation. Asterisks mark statistical significance (*p* < 0.05)

Three high-throughput platforms (microarray, TaqMan Low Density Array and Illumina small RNA Sequencing) of miRNA expression were used to detect cell cycle dependent miRNA expression (Fig. 4, Panel a-d, Additional file 2: Figure S5). Among them, microarray (Fig. 4, Panel a and c) displayed the lowest dynamic range and was unable to detect miRNAs of altered expression between cell cycle phases in HDFa and NCI-H295R cells. TaqMan Low Density array (Fig. 4, Panel b) performed on RNA isolated from sorted NCI-H295R cells detected 8 miRNAs of altered expression between cell cycle phases (among which only hsa-miR-10b, hsa-miR-128a and hsa-miR-890 had fold change values exceeding 2), however qRT-PCR validation of selected miRNAs failed to confirm the results (Additional file 2: Figure S6). Among the three platforms used in our study, small RNA sequencing was found to have the largest dynamic range in detection of miRNA expression alterations (Fig. 4, Panel d and Additional file 2: Figure S5). Still, statistical analysis detected only 11 miRNAs with altered expression in HeLa cells, of which only four miRNAs (hsa-miR-146b, hsa-miR-577, hsa-miR-877 and hsa-miR-193b*) had FC > 2 expression change between cell cycle phases. QRT-PCR measurements, similar to that of TLDA validation attempt, failed to validate differential expression in NCI-H295R and HeLa cells (Additional file 2: Figures S5 and S6). For further validation four other miRNAs showing stable expression in different cell types and cell cycle phases based on the high-throughput data were selected for qRT-PCR control analysis, which confirmed the stable expression pattern (Additional file 2: Figure S6).

Among several cell cycle regulator miRNAs, members of the hsa-miR-16 family were found to display dynamic changes in expression between serum-starved G0 and actively proliferating state [23]. Therefore, we analyzed expression changes of the hsa-miR-16 family members: hsa-miR-16, hsa-miR-15a and hsa-miR-503 in our high-throughput data (Fig. 4, Panel a-d and Additional file 2: Figure S5, Panel a) and performed qRT-PCR analysis as well (Fig. 4, Panel e-g). In the case of hsa-miR-15a, small RNA sequencing detected two-fold alteration in expression in NCI-H295R and HeLa cells as well and qRT-PCR analysis further confirmed some small, but significant expression changes in all three cell types.

## Discussion

With the advent of high-throughput transcriptional profiling, the precise analysis of the cell cycle transcription program became possible. Time course gene expression data after various synchronization procedures were used for gene expression profiling on microarray platforms [4–6] which demonstrated the cell cycle transcription program in various cell types including untransformed primary [5, 6] and transformed cancer cells [4].

However, questions were raised on the applicability of synchronization procedures in the analysis of cell cycle dependent transcriptional profiling [7]. In fact, growth imbalance and unscheduled expression of key cell cycle factor cyclins were demonstrated due to synchronization procedures [8, 9]. Additionally, cells failed to retain their synchronization relatively soon after depriving the synchronization agent from growth medium [5, 10]. A rigorous set of criteria was introduced by Shedden and Cooper concerning the analysis of cell cycle dependent gene expression [7] and, therefore, novel methods satisfying these criteria were needed. Centrifugal elutriation [25] and cell cycle sort [26–28] are two synchronization-free methods satisfying the Shedden and Cooper criteria without perturbing the cell cycle machinery. However, to our knowledge no detailed study using these methods to determine cell cycle dependent gene expression in human cells has been published to date. Upon centrifugal elutriation, cells are differentiated upon the size of each cell [25], while during cell cycle sorting by FACS the amount of DNA in each cell is used for separation [27, 28]. Both methods achieved efficient separation of cell cycle phases [12, 13, 27–29]. Although proper segregation of cell cycle phases by cell cycle sort may be optimal in cells lacking aneuploidy, cells presenting aneuploidy may as well be introduced to cell cycle sort until proper further confirmation of cell cycle sort (detection of the different expression of key cell cycle regulators e.g. Tyr-15 phosphorylation of CDC-2 [24]) supports the results. In our results, dynamic changes of Tyr-15 phosphorylation of CDC-2 in HeLa show as great variability as in NCI-H295R cells, confirming successful segregation of cell cycle phases in HeLa cells as well.

Here, using the cell cycle sort method, we report a comparative analysis of the cell cycle dependent gene transcriptional profile of human untransformed primary (fibroblasts) and cancer (adrenocortical and cervical) cells. Functional bioinformatics analysis revealed cell cycle related molecular and cellular functions to be mostly concerned with these transcriptional alterations. As time-course gene expression data from previous synchronization-based studies regarding primary human fibroblasts [5] and HeLa cells [4] were available, we performed a re-analysis of these data to compare synchronization-based and cell cycle sorting-based results. Upon the significant correlation between our data and these earlier results, we conclude that data obtained from cell cycle sort experiments confirm earlier results demonstrating cell cycle dependent gene expression in human cells, as well as it satisfies the rigorous criteria described above [7].

Moreover, GO term analysis was performed to assess biological processes related to the HeLa cell cycle dependent transcriptional program. The overlap between HeLa cell cycle sort and HeLa synchronization experiments showed a robust enrichment of cell cycle-related GO terms, cross-validating the key players of the cell cycle dependent transcriptional program. Additionally, cell cycle-related biological processes were enriched in both the unique to HeLa cell cycle SORT and the unique to HeLa synchonization gene lists. However, interestingly, the majority of GO terms detected in the unique HeLa synchronization gene list were absent from the overlap gene list, indicating some specific mechanisms related to synchronization procedures. The specific presence of ”response to DNA damage stimulus” and ”cellular response to stress” and the induction of ”DNA repair” GO terms confirms the replication stress as a consequence of synchronization procedures [8, 9, 30]. These analyses further confirm that our synhronization-free method of cell cycle sort charactherises more specifically the cell cycle of unperturbed cells than the synchronization-based methods.

We also investigated the eventual difference of cell cycle regulated transcriptional program in human untransformed and cancer cells. Whitfield et al. demonstrated that genes exhibiting cell cycle regulated expression were overexpressed in malignant tumors reflecting the malignancy signature of neoplasms [4]. This was explained by the fact that tumors contain more cycling cells [4]. Cell cycle dynamics alter disproportionally during malignant transformation [31]: activation of oncogenes *HRAS*, *SRC*, *MYC*, *CCND1*, *CCNE* [32–34], and loss of tumor suppressor genes as *PTEN* [35] shortens G1 phase [31], while loss of key M phase regulators *LZTS1* and *LATS2* results in M phase shortening [31, 36, 37]. These alterations lead to a relatively larger portion of cells residing in S and G2 phases. Additionally, certain gene clusters were confirmed to exhibit cell cycle dependent expression in either primary untransformed or transformed cancer cells [5], differentiating cells upon malignant transformation.

**Fig. 5:**
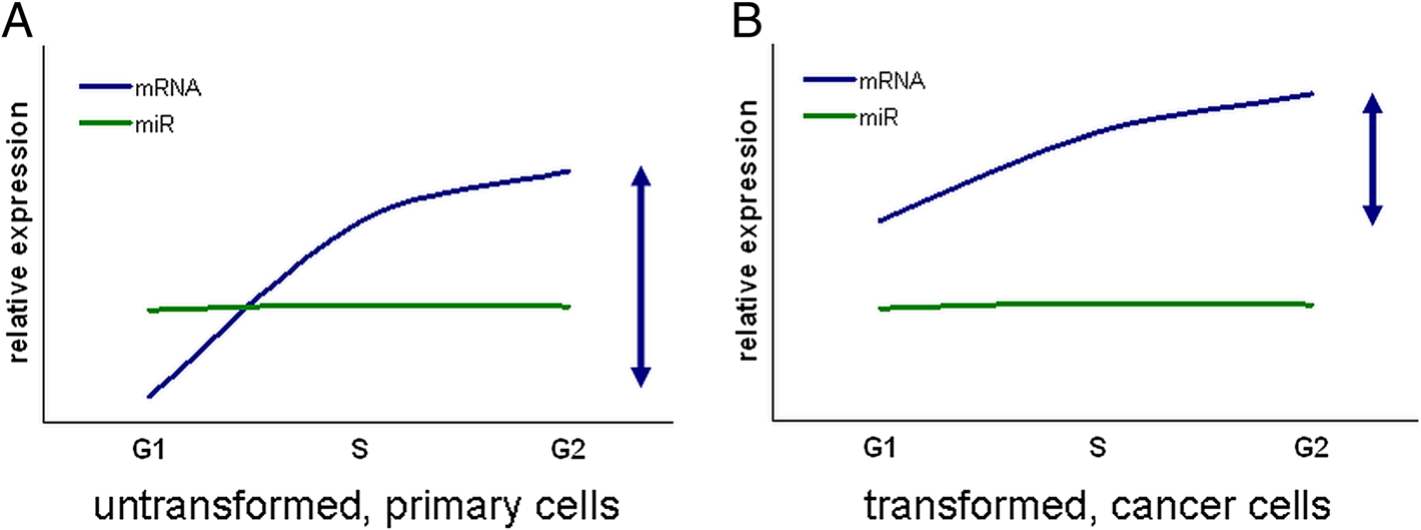
Schematic presentation of the hypothesis concerning expression dynamics of mRNAs and miRNAs during the cell cycle phases in primary untransformed and cancer cells. Relative expression changes in G1, S and G2 phases of a representative cell cycle gene and miRNA in untransformed primary (Panel **a**) and transformed cancer (Panel **b**) cells. Relative expression changes of mRNAs exhibiting cell cycle dependent expression (dark blue) and cell cycle associated miRNAs (green) are shown. Blue double arrows mark the amplitude of dynamic expression range in cell cycle phases. Note the different phase-specific expression values and the different dynamic expression range in the two cell types. Upon our results, genes exhibiting cell cycle dependent expression profile in untransformed cells are characterized with lower expression levels throughout the cell cycle, however they possess greater variance in expression levels than in transformed, cancer cells. MiRNAs, however, do not display cell cycle dependent expression

Our results contribute to the notion of different transcriptional regulation in untransformed and cancer cells. Since we have analyzed only three human cell types of different tissue origin, we can not draw a definitive conclusion universal to the cell cycle effects of malignant transformation. However, based on our analysis we may hypothesize that genes displaying universal cell cycle dependent expression in untransformed and cancer cells display altered expression in each phase and dynamic changes of different amplitude (Fig. 5). MRNA expression was found to be higher in G1, S and G2 phases as well, therefore, in addition to altered cell cycle distribution, basal, phase-independent up-regulation of these cell cycle genes may as well constitute to the well observed higher expression in malignant cancers. Dynamic mRNA expression differences between G1 and S phases were of greater amplitude in untransformed primary cells than in cells undergoing malignant transformation. This may be explained by the longer and more tightly controlled G1 phase and G1/S transition observed in untransformed, primary cells [31], as it reflects the more precisely regulated cell cycle machinery in untransformed cells. Moreover, MYC amplification stimulates E2F expression in cancer cells, facilitating the commitment to cell division [38, 39]. This facilitated regulation of the G1/S transition may as well contribute to smaller expression changes of the cell cycle dependent transcriptional program.

MiRNAs have a well established role in the regulation of the cell cycle [19]. Oncogenic (onco-miRs) and tumor-suppressor (TS-miRs) miRNAs were confirmed as modifiers of key cell cycle agents, accelerating or decelerating cell cycle progression [40, 41]. Long-term miRNA-mediated cell cycle changes contribute to malignant transformation in a variety of neoplasms [19, 42–46]. Additionally, the role of miRNA-mediated regulation has been confirmed in the transition from quiescent state to actively proliferating state [19, 23]. In particular, mitogenic stimuli enhances cell cycle progression by stimulating key transcriptional factors of the E2F family, which in turn enhances members of the hsa-let-7 and hsa-miR-16 families [17, 22, 23]. These well-known TS-miRs target key cell cycle cyclins as cyclin E, fine-tuning the proper cell cycle progression [17]. However, the proposed cell cycle dependent miRNA expression pattern [19] has not been thoroughly investigated, as according to our knowledge only one synchronization study identifying some cell cycle regulated miRNAs has been published to date [47].

Our study aiming to detect miRNA expression changes between cell cycle phases included the application of miRNA microarray, qPCR-based TLDA and Illumina small RNA sequencing. Microarrays are widely used for high throughput miRNA profiling and produce results which can be validated in high percentage by qPCR [48]. However, obtaining negative results prompted us for further analysis using qPCR-based TLDA and Illumina small RNA sequencing. The latter approach has larger dynamic range of detection allowing us to successfully detect smaller, but significant alterations [49, 50]. QPCR-based TLDA results however can be most successfully validated by single tube individual miRNA-specific qPCR as primer sequences used in TLDA does not differ.

Analysis of the three human cells (two cancer cell lines and one primary cell) on three high-throughput miRNA expression platform in our study revealed that miRNA expression profile throughout the cell cycle phases was quite stable (Fig. 5). Surprisingly, our systematic study using multiple high-throughput platforms indicated the lack of validable cell cycle dependent miRNA expression, and also showed that fold change differences are of small amplitude, especially in the light of the robust and explicit changes observed in mRNA expressions of the very same cell stage samples. More than 50 % of miRNA genes are located in cancer-associated genomic regions or in fragile sites, being continuously downregulated or deleted in cancers [40, 51]. Therefore, the loss of TS-miRs and the activation of onco-miRs are specifically involved in the long-term malignant transformation. Moreover, with the loss of genetic regions containing miRNA genes, the possibility of their dynamic regulatory functions throughout the cell cycle is lost as well [40, 51]. Additionally, miRNA-dependent gene regulation was found to be a much slower process than previously thought, due to certain bottlenecks related to the complex biogenesis and maturation processes or delays of miRNAs loading into Argonaute proteins [52]. Accelerated turnover was proposed to be necessary for certain miRNAs to be possibly involved in dynamic cell cycle regulation [52]. Such accelerated turnover has been confirmed in the case of the hsa-miR-16 family [23]. This family has been identified as a cluster of TS-miRs [19], downregulated in various types of cancers [19, 42–44] and associated with quiescent state [23]. Our results indicated some minor miRNA expression changes, especially in the case of hsa-miR-15a, however, expression changes were not fully congruent in the three cell types studied, suggesting that the well known, cell type specific expression of miRNAs may contribute to this phenomenon.

Finally, it is of utmost importance to address the limitations of our study. Firstly, upon the analysis of three human cell types of different tissue origin we can not draw a general conclusion concerning the difference of the expression dynamics of the universal cell cycle genes. Secondly, although different culture conditions may have some effect on the observed cell cycle differences, our method has much less effects on cell cycle alterations compared to previous serum shock-based or inhibitive synchronization-based processes.

## Conclusions

In conclusion, successful utilization of cell cycle sort as a novel method for the analysis of cell cycle transcriptional program in our study confirmed the previously identified cell cycle transcriptional regulation. Different phase-dependent and phase-independent mRNA expression dynamics of cell cycle genes in human untransformed and cancer cells were revealed, reflecting the altered cell cycle machinery in cancer cells at the transcriptional level. Perhaps more interestingly, the application of various high-throughput platforms (microarray, TLDA, Small RNA Sequencing) for miRNA profiling showed that miRNA expression dynamics are unaltered during the active cell cycle at the G1/S and S/G2 transitions.

## Methods

### Cell culture

Human adrenocortical cancer cell line NCI-H295R and human cervical cancer cell line HeLa were obtained from the American Type Culture Collection (ATCC), while human dermal fibroblast (HDFa) cells were obtained from Gibco (Life Technologies). NCI-H295R cells were cultured in Dulbecco’s modified Eagle’s medium/Nutrient Mixture F-12 Ham (DMEM: F12) supplemented with 6.25 ng/ml insulin, 6.25 ng/ml transferrin, 6.25 ng/ml sodium selenite, 1.25 mg/ml bovine serum albumine, 5.35 ng/ml linoleic acid, 1 % HEPES, 1 % Penicillin-Streptomycin, 2.5 % L-glutamine (Sigma-Aldrich Chemical Co.) and 2.5 % Nu-Serum (BD Biosciences). HeLa cells were cultured in Dulbecco’s modified Eagle’s medium/Nutrient Mixture F-12 Ham (DMEM: F12, Sigma-Aldrich Chemical Co.) supplemented with 10 % fetal bovine serum (Gibco by Life Technologies) and 1 % antibiotic-antimycotic solution (Sigma-Aldrich Chemical Co.). HDFa cells were cultured in Medium 106 supplemented with low serum growth supplement (LSGS, Gibco by Life Technologies). All cells were cultured at 37 °C in a humidified 5 % CO_2_ atmosphere.

### DNA content based fluorescence activated cell sorting (FACS)

HDFa, NCI-H295R and HeLa cells were cultured in 150 cm^2^ cell culture flasks until 90 % confluency. Cells were trypsinized, washed, resuspended in complete medium and counted. Vybrant DyeCycle Orange (Molecular Probes by Life Technologies) was used to stain genomic DNA stoichiometrically in living cells (approximate fluorescence excitation and emission maxima were 519 nm and 563 nm, respectively), and was added in 1:500 dilution to 1 × 10^6^ cells/ml cell suspension. After incubation at 37 °C for 30 min, protected from light, cells were centrifuged at 1000 rpm for 10 min and were resuspended in the sort medium (Hank’s Balanced Salt Solution without Ca^2+^ and Mg^2+^, containing 2 % fetal calf serum). FACSAria III cell sorter (Becton-Dickinson, Franklin Lakes, NJ, USA) was used for cell cycle analysis and sorting using 488 nm Argon laser. The fluorescence emission of Vybrant DyeCycle Orange was separated by a 556 longpass filter and detected through a 585/42 bandpass filter. At least 100,000 events were collected for analysis. Upon cell cycle analysis, cell populations resembling G1, S and G2 phases were gated according to cellular DNA quantity. Sorting did not exceed 30 min and all sorted populations were validated by flow cytometry analysis. Data were analyzed by BD FACSDiva v6.1.3 software (BD Biosciences, San Jose, CA, USA). Thereafter, cells were centrifuged, washed with ice-cold PBS and resuspended in QIAzol lysis reagent (Qiagen) or Western blot lysis buffer for subsequent RNA or protein isolation, respectively. Until RNA isolation or Western blot, samples were stored at −80 °C. Optimization from the protocol supplied by the manufacturer included the use of sort medium, concentration of the cell suspension before FACS analysis, respecting an upper time limit for sorting and immediate FACS reanalysis upon every cell cycle-sorted population.

### Protein isolation and Western blot

Samples were thawed on ice, sonicated with ultrasound and incubated on ice for 30 min. Thereafter, samples were centrifuged at 13000 rpm and 2 °C for 15 min. Protein concentration was determined according by Bradford method using Varioskan Flash spectral scanning reader (Thermo Scientific) [53]. Optical density was determined at 595 nm. Samples were mixed with β-mercaptoethanol containing Laemli buffer and were incubated at 99 °C for 5 min. Thereafter, equal amount of samples were loaded on a 10 % polyacrylamide gel and electrophoresis was conducted on a Mini Protean electrophoresis equipment (Bio-Rad). Overnight blotting at 4 °C was performed to transfer proteins to a PVDF membrane (Millipore, Billerica, MA). Blotting efficiency was determined by Ponceau staining. Membranes were blocked with 5 % non-fat dry milk in TBS for 60 min at room temperature, and were incubated with primary phospho-CDC-2 (Tyr15) antibody (Cell Signaling Technology, cat. No.: 9111, dilution: 1:500) at 4 °C for 16 h. Thereafter, membranes were washed 5 times with 0.05 % Tween-20 containing TBS, and were incubated with secondary antibody (Cell Signaling Technology, cat. No.: 7074, dilution: 1:2000). All antibodies were diluted in 1 % non-fat dry milk containing TBS. After exposure to SuperSignal West Pico Chemiluminescent Substrate (Thermo Scientific), signals were visualized by Kodak Image Station 4000MM Digital Imaging System. Thereafter membranes were stripped with mild stripping buffer (0.2 M glycine, 0.1 % sodium dodecyl sulfate, 0.1 % Tween-20, pH = 2.2) by gentle agitation for 45 min at room temperature, and were blocked again for subsequent detection of loading control β-actin (Cell Signaling Technology, cat. No.: 4967, dilution: 1:2000). Membrane blocking, antibody incubations and signal detection were carried out exactly as in the case of phospho-CDC-2 detection. Densitometry of the detected bands was performed by Kodak Image Station. β-actin was used as loading control.

### RNA isolation, messenger RNA profiling and validation by qRT-PCR

Total RNA was isolated using miRNeasy Mini Kit (Qiagen), according to the manufacturer’s instructions and was eluted in 50 uL nuclease-free water (Qiagen). RNA concentration and integrity was determined by the Agilent Bioanalyzer 2100 system (Agilent Technologies, Additional file 2: Figure S1, Additional file 1: Table S1).

### High throughput profiling of gene and miRNA expression

#### Gene expression profiling

Gene expression profiling was performed on 100 ng RNA isolated from sorted G1, S and G2 phases of HDFa, NCI-H295R and HeLa cells. In all, 24 samples (2 or 3 samples of each phase) were analyzed using Agilent whole human genome 4x44K microarray slides (Agilent Technologies) following the manufacturer’s protocol [54].

### miRNA expression profiling using microarray

MiRNA expression profiling was performed on 100 ng RNA isolated from sorted G1, S and G2 phases of HDFa and NCI-H295R cells. In all, 16 samples (2 or 3 samples of each phase) were analyzed. The miRNA expression profiling using microarray followed the manufacturer’s protocol [48]. Total RNA was labeled with Cy3 and amplified using Low Input Quick Amp Labeling Kit according to the manufacturer’s instructions. After RNA purification, labeled RNA was hybridized to Agilent 8 × 15 K Human miRNA Microarray Release 12.0. slides (Agilent Technologies), according to the manufacturer’s instructions. After washing, array scanning and feature extraction was performed by Agilent DNA Microarray Scanner and Feature Extraction Software 11.0.1.

### miRNA expression profiling using TaqMan Low Density Array (TLDA)

RNA isolated from two samples of G1 and three samples of S and G2 phase-sorted NCI-H295R cells were studied using TaqMan Low Density Array (TLDA) cards, according to the manufacturer’s instructions. The miRNA expression profiling using TLDA was performed as previously reported [55]. 30 ng of total RNA was reverse transcribed and pre-amplified using Megaplex RT primer pool A and B and Megaplex PreAmp primers, respectively. Quantitative real-time PCR were carried out in TaqMan Human MicroRNA Array A and B on a 7900HT Real time PCR System (Applied Biosystems by Life Technologies).

### miRNA expression profiling using Illumina small RNA sequencing

Two samples of each cell cycle phase of HeLa cells (six samples) and one sample of each cell cycle phase of pooled sorted NCI-H295R cells (threee samples) were analyzed. Small RNA sequencing was performed at BGI using Illumina Small RNA Sequencing Platform. For library preparation TruSeq Small RNA library preparation kit (Illumina, San Diego, California) was used. Sequencing was performed by SE50 with Illumina HiSeq2000, and 10 Mb clean reads were analyzed followed by routine algorithms (BGI Tech Solutions, Tai Po, Hong Kong).

### qRT-PCR validation

For the gene expression qRT-PCR experiments, 30 ng of total RNA was reverse transcibed using SuperScript VILO cDNA synthesis kit according to the manufacturer’s instructions (Applied Biosystems by Life Technologies). Gene expression was quantified using predesigned Taqman probes (Additional file 1: Table S2, Applied Biosystems by Life Technologies) on a 7500 Fast Real-time PCR system (Applied Biosystems by Life Technologies). Gene expression data were normalized to the relative expression of ACTB.

For the miRNA expression qRT-PCR experiments, 5 ng of total RNA was reverse transcribed and quantified using TaqMan microRNA reverse transcription kit (Applied Biosystems by Life Technologies) and predesigned TaqMan probes (Additional file 1: Table S2, Applied Biosystems by Life Technologies) on a 7500 Fast Real-time PCR system (Applied Biosystems by Life Technologies). MiRNA expression data were normalized to the relative expression of RNU48.

All measurements were performed in triplicate (three biological, two technical replicates). Expression level was calculated by the ΔCt(S-phase) – ΔCt(G1-phase) and the ΔCt(G2-phase) – ΔCt(G1-phase) (ΔΔCt) methods.

### Pathway analysis

Ingenuity Pathway Analysis (IPA, Ingenuity Systems) was used to detect molecular and cellular functions altered between cell cycle phases. Δ(G2-G1) gene expression changes of significantly differently expressed genes (NCI-H295R and HeLa) or genes with fold change > 2 expression (HDFa) were subjected to IPA core analysis.

### Microarray data from former studies

Two former microarray studies identifying cell cycle dependent expression of mRNA transcripts in human primary fibroblasts [5] and HeLa cells [4] using synchronization based procedures were selected. Processed data from these experiments were downloaded from http://genome-www.stanford.edu/Human-CellCycle/HeLa/ [4] and from the European Bioinformatics Institute Array Express database (http://www.ebi.ac.uk/arrayexpress/experiments/E-TABM-263/) [5] and were re-analyzed [4, 5]. Upon published FACS analysis data, time points with highest levels of synchronous populations of each cell cycle phase were chosen to represent G1, S and G2 phases, respectively. Difference in gene expression between phases was calculated upon difference of normalized expression of a certain gene between time points representing each phase.

In these comparisons only those gene expression alterations were used where the cell cycle sort indicated cell cycle dependent gene expression changes (FC > 2 genes of HDFa and significant genes of HeLa experiment).

### Gene ontology term analysis

Gene Ontology (GO) term analysis was performed to detect biological processes with enriched genes in the HeLa cell cycle transcriptional program. The online functional annotation tool of DAVID Bioinformatics Resources version 6.7 (https://david.ncifcrf.gov/) with Gene Ontology for biological processes (category: GOTERM_BP_FAT) was used. The input gene lists for the analysis were the genes unique to HeLa SORT experiment (HeLa SORT \ HeLa synchr), unique to HeLa synchronization experiment (HeLa synchr \ HeLa SORT) and the overlap between these two lists (HeLa SORT ∩ HeLa synchr). Bonferroni-corrected *p*-values < 0.05 were considered statistically significant [4].

### Analysis of gene expression levels and cell cycle dynamics in primary and cancer cells

Analysis of gene expression levels and cell cycle dynamics in different cell types were performed by investigating the changes of 127 genes found to be cell cycle dependently expressed in HDFa and HeLa cells. Upon combined normalization of all cell cycle sort-based gene expression microarrays, normalized intensity values in each cell type in each cell cycle phase were compared. For the analysis of the gene expression dynamism during cell cycle progression the absolute values of fold changes between cell cycle phases were calculated and were subjected to comparison between HDFa, NCI-H295R and HeLa cell types.

Results of qRT-PCR experiments in 10 (Additional file 1: Table S2) out of these 127 genes were also subjected to these analyses. ΔCt values normalized to *ACTB* expression and absolute values of fold changes in cell cycle phases were calculated and were compared in all cell types.

### Statistical analysis

Statistical analysis of the microarray data was performed by GeneSpring 12.6 (Agilent Technologies) software. Total signal normalization at the 75th percentile of raw signal values and baseline transformation at the median of all samples following Agilent’s recommendation were performed. Differently expressed genes between G1, S and G2 phases were detected by one-way ANOVA followed by Tukey’s Honestly Significant Difference post hoc test and Benjamini-Hochberg correction for multiple measurements.

ΔCt levels of individually measured mRNA and miRNA transcripts obtained by qRT-PCR measurements and subsequent normalization to housekeeping transcripts (ACTB or RNU48) were subjected to Students’ two sided independent samples *T*-test. Differences were analyzed between G1-S, S-G2 and G1-G2 phases, respectively. Center values shown are the average of replicate experiments.

On genes displaying cell cycle dependent expression revealed by cell cycle sort, Pearson’s correlation was used to calculate correlation between expression changes detected by different (cell cycle sort and various synchronization) methods.

Student’s two-sided paired samples *T*-test was used to detect difference in normalized expression of genes expressed in a cell cycle dependent manner between various cell types. Student’s two-sided independent samples *T*-test was used to detect difference in absolute values of fold change of cell cycle dependently expressed genes of various cell types. In all comparisons *p*-value <0.05 was considered statistically significant.

Statistical analysis for miRNA expression analysis of TLDA card was performed using Real-Time StatMinerTM software (Integromics, Granada, Spain). Expression level was calculated by the ΔΔCt method, and fold changes were obtained using the formula 2^-ΔΔCt^. Following quality control, expression levels were normalized to the geometric mean of all expressed miRNAs. One-way ANOVA was used to detect significantly altered expression. In all comparisons *p*-value <0.05 was considered statistically significant.

For identification of differentially expressed miRNAs of Small RNA Sequencing experiments edgeR package version 3.8.6 in R was used. Alignment to MirBase version 21.0 mature miRNA database was performed on reads longer than 18 nucleotides with maximum 1 mismatch. The input data for edgeR package were the pair of phases (G1-S, S-G2, G1-G2) with two samples for each phases. The classical exact *T*-Test and TMM normalization were applied. Benjamini and Hochberg’s algorithm was used to control the false discovery rate (FDR). The difference was statistically significant when both the *p*-value and the FDR was <0.05.

## Additional files

Additional file 1: Table S1. Characterization of cell cycle sorted cells and isolated RNA quantity in various cell types. Purity of cell cycle sort was determined by re-analyzing the sorted populations by FACS analysis: percentage was determined by the portion of cells residing in the gate previously designated for a certain cell cycle phase. Data of four replicate experiments. Data are given as × 1000 cells (sorted cells) and are shown as mean ± standard deviation. **Table S2.** Name and details of primers used for mRNA and miRNA expression qRT-PCR measurements. mRNA primers (Panel A, cat. No: 4331182) and miRNA primers (Panel B, cat. No: 4427975). All primers were from Applied Biosystems by Life Technologies. **Table S3.** Normalized expression of differently expressed genes between cell cycle phases in various cell types. Normalized expression of genes with fold change > 2 between cell cycle phases detected in HDFa cells (Panel A). Normalized expression of significantly differently expressed genes in cell cycle phases detected in NCI-H295R (Panel B) and HeLa (Panel C) cells. Note: For Panel B and C, genes are listed in the manner as shown in the heat map (Fig. 2, panel A and B, respectively). **Table S4.** List of genes shown on Venn diagram (Fig. 3, panel a). Genes are marked with “1” if being found cycling by either method (HDFa SORT, PF synchr, HeLa SORT, HeLa synchr). Gene IDs are Gene Symbols, if available or probe IDs. **Table S5.** List of HeLa cell cycle genes being present in enriched GO terms. Table 1 presents GO terms which are enriched in gene lists unique to HeLa SORT, HeLa synchronization experiments and the overlap beween HeLa SORT and synchronization lists. Gene symbols of genes being present in the gene lists and the enriched GO terms are shown here (HeLa SORT \ HeLa synchr - Panel A; HeLa synchr \ HeLa SORT – Panel B; HeLa SORT ∩ HeLa synchr – Panel C). (XLS 743 kb) Additional file 2: Figure S1. Quality and integrity of RNA isolated from cell cycle sorted populations of G1, S and G2 phases of HDFa, NCI-H295R and HeLa cells. Isolated RNA was analyzed on Agilent Bioanalyzer 2100 System. Representative results of G1, S and G2 populations sorted from HDFa (Panel A), NCI-H295R (Panel B) and HeLa (Panel C) cells are shown. RNA integrity number (RIN) was calculated if RNA concentration exceeded 10 ng/uL, therefore, in samples with lower RNA concentration RIN is not shown. **Figure S2.** Supplementary functional bioinformatics analysis of molecular and cellular functions concerned by gene expression alterations in cell cycle phases. Panel A: HDFa, Panel B: NCI-H295R, Panel C: HeLa. In Fig. 2, panels f-h only the five most significantly concerned networks are shown. Additional molecular and cellular functions are shown here. **Figure S3.** Pearson’s correlation analysis of gene expression changes in cell cycle phases of cell cycle sort and synchronization method in primary fibroblasts. Data of synchronization experiments: [5]. Synchronization methods: SS – serum starvation, SST – serum starvation followed by thymidine block. Correlation coefficients are shown. Asterisks mark statistical significance (*p* < 0.05). **Figure S4.** Pearson’s correlation analysis of gene expression changes in cell cycle phases of cell cycle sort and synchronization method in HeLa cells. Data of synchronization experiments: [4]. Synchronization methods: DT – double thymidine block, TN – thymidine followed by nocodazole block. Correlation coefficients are shown. Asterisks mark statistical significance (*p* < 0.05). **Figure S5.** Illumina Small RNA Sequencing and qRT-PCR measuremnts of cell cycle sorted NCI-H295R cells. Panel A: Fold change (log2) of miRNA expression in S/G1, G2/S and G2/G1 phases observed by Illumina Small RNA Sequencing. One pooled sample of each cell cycle phase was sequenced and, therefore, no statistical analysis has been performed. Grey background corresponds to fold change ≤ 2 between cell cycle phases. Colored circles represent some members of the hsa-miR-16 family, if available. Colored triangles represent two chosen miRNAs with FC > 2 expression between G2/G1 phases chosen to validate high-throughput data. Panel B: QRT-PCR measurements of 4 miRNAs chosen to validate Illumina Small RNA Sequencing data. Hsa-miR-202 and hsa-miR-132 were chosen upon FC > 2 expression in cell cycle phases. Hsa-miR-24-2* and hsa-miR-577 were chosen as G1 phase lacked miRNA expression, while in G2 phase miRNA expression was detectable. However, neither statistical examination detected significant difference (*p* < 0.05). Error bars show standard deviation. **Figure S6.** QRT-PCR measurements of miRNAs chosen to validate high-throughput data in HDFa, NCI-H295R and HeLa cells. Hsa-miR-10b, hsa-miR-128a and hsa-let-7 g were chosen upon TLDA screening of NCI-H295R cells. Hsa-let-7a, hsa-let-7e and hsa-let-7f were chosen upon Small RNA Sequencing of cell cycle sorted HeLa cells. MicroRNAs of stable expression (hsa-let-7i, hsa-miR-21, hsa-miR-22 and hsa-miR-222) in all three cell types and on all platforms were chosen to validate stable expression. Data from HDFa cells (Panel A), NCI-H295R cells (Panel B) and HeLa cells (Panel C). Additionally, hsa-miR-577 was also measured in HeLa cells, but it showed no cell cycle dependent expression pattern (Panel D). Error bars show standard deviation. Neither statistical examination detected significant difference (*p* < 0.05). (TIF 636 kb)
