# Supplementary figures and images for: Fluorescence activated cell sorting followed by small RNA sequencing reveals stable microRNA expression during cell cycle progression
Source: BMC Genomics. 2016 May 27;17:412. doi: 10.1186/s12864-016-2747-6 (PMC4884355; doi:10.1186/s12864-016-2747-6)

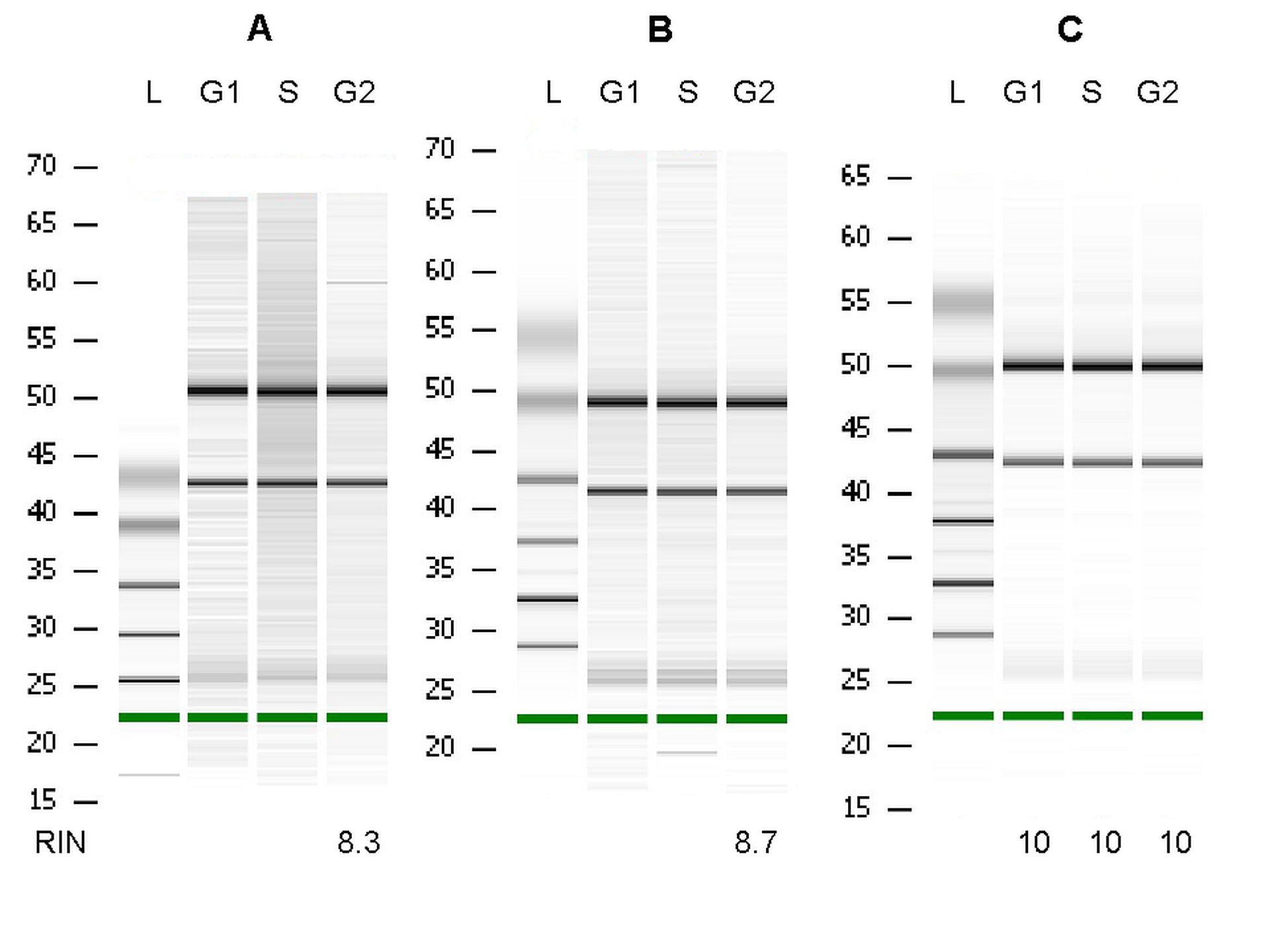

Supplement: Additional file 2: Figure S1. — Quality and integrity of RNA isolated from cell cycle sorted populations of G1, S and G2 phases of HDFa, NCI-H295R and HeLa cells. Isolated RNA was analyzed on Agilent Bioanalyzer 2100 System. Representative results of G1, S and G2 populations sorted from HDFa (Panel A), NCI-H295R (Panel B) and HeLa (Panel C) cells are shown. RNA integrity number (RIN) was calculated if RNA concentration exceeded 10 ng/uL, therefore, in samples with lower RNA concentration RIN is not shown. Figure S2. Supplementary functional bioinformatics analysis of molecular and cellular functions concerned by gene expression alterations in cell cycle phases. Panel A: HDFa, Panel B: NCI-H295R, Panel C: HeLa. In Fig. 2, panels f-h only the five most significantly concerned networks are shown. Additional molecular and cellular functions are shown here. Figure S3. Pearson’s correlation analysis of gene expression changes in cell cycle phases of cell cycle sort and synchronization method in primary fibroblasts. Data of synchronization experiments: [5]. Synchronization methods: SS – serum starvation, SST – serum starvation followed by thymidine block. Correlation coefficients are shown. Asterisks mark statistical significance (p < 0.05). Figure S4. Pearson’s correlation analysis of gene expression changes in cell cycle phases of cell cycle sort and synchronization method in HeLa cells. Data of synchronization experiments: [4]. Synchronization methods: DT – double thymidine block, TN – thymidine followed by nocodazole block. Correlation coefficients are shown. Asterisks mark statistical significance (p < 0.05). Figure S5. Illumina Small RNA Sequencing and qRT-PCR measuremnts of cell cycle sorted NCI-H295R cells. Panel A: Fold change (log2) of miRNA expression in S/G1, G2/S and G2/G1 phases observed by Illumina Small RNA Sequencing. One pooled sample of each cell cycle phase was sequenced and, therefore, no statistical analysis has been performed. Grey background corresponds to fold change ≤ 2 between [file 12864_2016_2747_MOESM2_ESM.tif]
